# Identification of New Biomarkers of Posturo-Locomotor Instability in a Rodent Model of Vestibular Pathology

**DOI:** 10.3389/fneur.2020.00470

**Published:** 2020-05-29

**Authors:** Emna Marouane, Guillaume Rastoldo, Nada El Mahmoudi, David Péricat, Christian Chabbert, Vincent Artzner, Brahim Tighilet

**Affiliations:** ^1^Aix Marseille Université-CNRS, Laboratoire de Neurosciences Sensorielles et Cognitives, LNSC UMR 7260, Equipe Physiopathologie et Thérapie des Désordres Vestibulaires, Marseille, France; ^2^BIOSEB SAS, Vitrolles, France

**Keywords:** vestibular compensation, posturo-locomotor instability, vestibular syndrome, automated analyses method, rodent model of vestibular pathology

## Abstract

The vestibular system plays a crucial role in maintaining postural balance. Unilateral vestibular lesions result in a typical syndrome characterized by postural imbalance, altered locomotor patterns and gaze stabilization, as well as cognitive and neurovegetative disorders. One of the main difficulties encountered in the development of new anti-vertigo drugs is the lack of sensitivity in the evaluation of this syndrome. Qualitative assessments of the vestibular syndrome have been developed, but methods of conducting quantitative evaluations are critically lacking. Recently, assessments with a dynamic weight-bearing device (DWB®, Bioseb) revealed postural alterations in rats subjected to unilateral vestibular neurectomy (UVN). Our team is evaluating a new version of this device capable of quantifying additional parameters of postural and locomotor equilibrium. The objective of this study was to use this device to assess these new posturo-locomotor parameters in a rat model of a vestibular pathology. The biomarkers measured by this device are as follows: the barycenter, the support surface and the weight distribution of the rats when they were moving or stationary. Before UVN, the rats showed a symmetric distribution of their weight along the lateral axis. In the acute phase after UVN on the left side, the rats distributed more weight on the right side than on the left side and then distributed more weight on the left side. These results corroborate those presented in our previous study. The support surface of the rats increased between 1 day and 30 days after UVN, and the barycenter distribution reflected the weight distribution. In addition, our results show smaller changes in the weight distributions when the animals are moving compared with when they are stationary in the acute phase after UVN. This study provides new information on the static and dynamic postural balance patterns observed after unilateral vestibular loss in rats. These data are relevant because they objectively quantify the posturo-locomotor component of vestibular syndrome as well as the compensatory strategies used after vestibular loss. These results may guide the development of rehabilitation protocols for vestibular patients and the validation of pharmacological compounds favoring the restoration of equilibrium.

## Introduction

**Figure 1:**
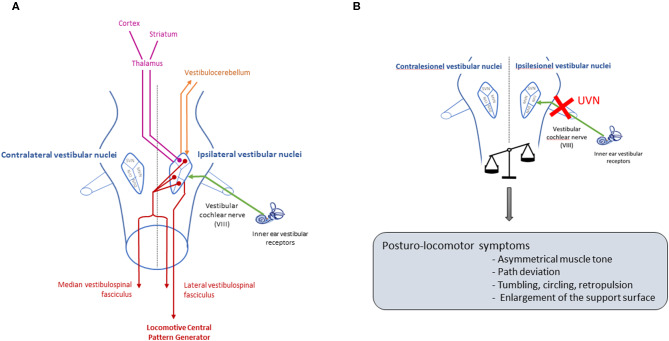
Pathways involved in posturo-locomotor function of the vestibular system, and deficits after unilateral vestibular neurectomy. **(A)** The vestibular nerve transmits information from peripheral vestibular receptors to the four vestibular nuclei located in the brain stem [medial vestibular nucleus (MVN), lateral vestibular nucleus (LVN), superior vestibular nucleus (SVN) and inferior vestibular nucleus (DVN)]. The vestibular nuclei then project toward various structures involved in the control of posturo-locomotor function: the spinal cord (17), the cerebellum (18), the striatum (19) and different cortical zones such as the motor cortex (20). The medial vestibulospinal fasciculus sends ipsi and contralateral projections to motoneurons of the neck muscles, allowing head stabilization, while the lateral vestibulospinal fasciculus projects ipsilaterally to the trunk and leg muscles, allowing anti-gravitary muscle tone, and to the locomotor CPG responsible for automatic locomotion coordination. The vestibular nuclei receive and transmit information to the vestibulocerebellum, involved in posture control, and via a thalamic relay, the vestibular nuclei project toward the striatum which is involved in locomotion control. The vestibular nuclei therefore constitute a central relay for the control of posture and locomotion, and a unilateral vestibular loss that induces electrophysiological imbalance between the homologous vestibular nuclei will generate a characteristic posturo-locomotor syndrome that affects each level of the neural network described above. **(B)** UVN will cause an electrophysiological asymmetry between homologous vestibular nuclei (21). Due to all the pathways previously described, this electrophysiological asymmetry will lead to various posture-locomotor symptoms.

The vestibular system is a sensory-motor system that plays a crucial role in postural control, locomotion, gaze stabilization and space orientation (1) (Figure 1A). Unilateral damage of this system leads to a vestibular syndrome involving posturo-locomotor, oculomotor, perceptual-cognitive and vegetative disorders (Figure 1B), which have a significant impact on patients' daily lives. The prevalence of this syndrome is high: an epidemiological study recently conducted on data from 70,000,000 patients in Germany estimated that the prevalence is 6.5% (2). However, preclinical studies lack reliable methods of evaluation to precisely quantify the vestibular syndrome, its evolution over time and the effect of different therapies on the kinetics of individuals with vestibular syndrome (3). In a rodent model, the consequences of unilateral vestibular injury are essentially assessed qualitatively by various scales that address several vestibular symptoms (e.g., circling, tumbling, retropulsion, or head-tilting) (4–7). These different scales describe the overall kinetics of the syndrome, and the disorders are most severe during the first 3 days after injury (a post-lesional critical period) and then decrease in severity until baseline values are restored (vestibular compensation) [(8) for review]. However, these scales remain subjective; it is therefore necessary to design new evaluation paradigms for the objective and parametric quantification of the vestibular syndrome. For several years, in human clinical practice, posturography has been commonly used for the assessment of postural vestibular disorders (9). The utilization of force platforms that record the movements of the patient's center of pressure and provide an estimate of the energy required for postural stabilization has proven to be a particularly sensitive and relevant diagnostic tool for the vestibular syndrome (10–13). Under different experimental conditions (static, dynamic, open and closed eyes), posturography allows a global evaluation of postural vestibular deficits. It is also a valuable tool for evaluating the effects of different vestibular therapies (pharmacological and rehabilitative) on the quality and timing of postural functional recovery (14).

This study aimed to identify new parameters to objectively quantify posturo-locomotor deficits in our rat model of unilateral vestibular neurectomy (UVN).

The first quantitative study conducted on the vestibular syndrome in rodents is relatively recent (6). The use of a weight distribution evaluation device (DWB®, Bioseb) to identify the bearing forces of the different limbs of animals has revealed severe alterations in the weight distributions in rats that have undergone unilateral vestibular neurectomy (UVN). With an advanced version of this device (DWB2®, Bioseb), we can differentiate static and dynamic vestibular behavior in our animal model and extract new data, such as the coordinates of the animal's limbs, at any time. To migrate from an evaluation that is essentially qualitative, in this study, we designed a new method of quantitative analysis based on parametric measurements. For the first time, we have demonstrated a postural imbalance following a vestibular lesion using parameters similar to those used in human clinical practice, such as plots of the position of the animal's barycenter under static condition (statokinesiograms), the surfaces of the 90% confidence ellipses of these statokinesiograms (15), the speed of displacement of the barycenter, and the speed of the barycenter as a function of the sway area (SFA: Speed as a Function of the Area), which yields estimates of the energy used for postural stabilization (16). Other behavioral phenotype characteristics of unilateral vestibular injuries were analyzed: the weight distribution on the medial lateral axis, the time spent by the animal leaning on its abdomen, and the number of laps performed during the circling periods. These data are consistent with those from our previous studies (6) and provide new information on the postural equilibrium pattern observed after vestibular loss in rats.

This parametric approach yields improved sensitivity in the evaluation of posturo-locomotor deficits following unilateral vestibular lesions. It will likely become an essential preclinical tool that is used to test the effectiveness of antivertiginous compounds or rehabilitation protocols on posturo-locomotor function recovery after vestibular loss.

## Materials and Methods

### Animals

The experiments were performed on 16 Long Evans male rats of 10–12 weeks old (250/300 g) originating from our own breeding, from parents arising from Charles River (St Germain sur l'Arbresle, France). All experiments were performed in accordance with the National Institutes of Health's Guide for Care and Use of Laboratory Animals (NIH Publication no. 80–23) revised in 1996 for the UK Animals (Scientific Procedures) Act of 1986 and associated guidelines or the Policy on Ethics approved by the Society for Neuroscience in November 1989 and amended in November 1993 and under the veterinary's supervision and the National Ethical Committee's control (French Agriculture Ministry Authorization: B13-055-25). Present study was specifically approved by Neurosciences Ethic Committee N°71 from the French National Committee of animal experimentation. Every attempt was made to minimize both the number and the suffering of animals used in this experiment. The animals were housed in a large confined space with 12–12 h diurnal light variations with free access to water and food. They were housed at the Fédération 3C (Center Saint-Charles, Aix-Marseille University) animal facility.

The 16 animals were divided into two groups: SHAM group (*n* = 7) and UVN group (*n* = 9). See below for the details of procedures used for surgery.

### Study Design

**Figure 2:**
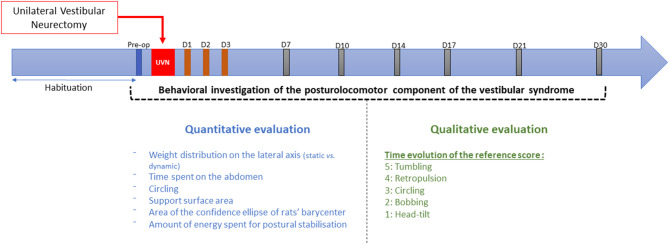
Study design. Details of the procedure used to evaluate quantitatively and qualitatively the vestibular syndrome before and after unilateral vestibular neurectomy. Behavioral investigation of the posturolocomotor component of the vestibular syndrome was made in a first preoperative session (serving as a reference value) and then at 1, 2, 3, 7, 10, 14, 17, 21, and 30 post-lesioned days (D). Two types of evaluation were carried: a first quantitative evaluation detailed in this study and a qualitative evaluation based on previous paper (5).

The behavioral investigations were carried out in 2 parts (Figure 2): a first quantitative evaluation of the syndrome with the DWB2®, and a second qualitative evaluation of the syndrome following the same scale as the one detailed in Péricat et al. (5). The rats were manipulated for 5 days before the preoperative session. During that period, a quantitative analysis of the postural parameters (reference values) was performed with the DWB2®. All the rats then underwent surgery before being assessed during the acute stage (post-lesion days 1, 2, and 3) and the vestibular syndrome compensated stage (post-lesion days 7, 10, 14, 17, 21, and 30).

### Surgery

The UVN was performed on nine rats following the surgical procedure previously reported in the literature (5). The rats were placed in the induction box and left for 5 min (isoflurane concentration 4%). Once they were deeply anesthetized, they were intubated and, during the surgery, the anesthesia was maintained at an isoflurane concentration of 3%. A tympanic bulla approach gave access to the vestibular nerve: the cervical muscular planes were dissected leading to the tympanic bulla, which was widely drilled to expose the stapedial artery and the promontory containing the cochlea. The cochlea was drilled, exposing the cochlear nerve. The cochlear nerve meatus was enlarged with a needle leading to the vestibulocochlear nerve, which was sectioned at its entry into the brainstem after aspiration of the Scarpa's ganglion. The wound was closed using a stapler. Before the animal awakened, a solution of Ringer Lactate (Virbac; 10 ml/kg) was injected subcutaneously to reduce the dehydration resulting from the rats' inability to drink normally due to the lesion. Buprenorphine (Buprecare® 0.05 mg/kg) was given 30 min before the surgery.

In another 7 sham rats, surgery was stopped at the opening of the tympanic bulla.

### Qualitative Evaluation of the Vestibular Syndrome

The vestibular syndrome induced in the rat after UVN is characterized by typical symptoms previously described in various species [rat UVN model: (5), mice UVN model: (4), cat UVN model: (7)]. These symptoms usually include tumbling, retropulsion, circling, bobbing and head-tilt which are all present in the acute phase of the syndrome and progressively disappear following vestibular compensation.

For this study, we used the same scale as the one previously reported (5). It is a cumulative scale where a score is assigned to each symptom, based on its severity (tumbling: 5, retropulsion: 4, circling: 3, bobbing: 2, head-tilt: 1).

### Quantitative Evaluation of the Vestibular Syndrome

#### Analysis Device

The second version of the dynamic weight-bearing (DWB2®) device (Bioseb, Vitrolles, France) was used to evaluate the postural instability of rodents following unilateral vestibular loss. This apparatus has previously been described for the assessment of postural instabilities in the same model of vestibular loss (6). It consists of a Plexiglas chamber (25 × 25 cm) with a floor covered by a 2,000 force sensors plate. The weight passed through each part of the body in contact with the ground was assessed automatically in each sensor at a sampling frequency of 30 Hz. A high frequency camera was directed at the side of the enclosure to assist with data analysis. Both the sensors and the camera were connected to a computer using the latest DWB2 software version available at the time (v2.0.60). The software was configured as follows: part of the animal body is detected if it activates at least 2 pixels and if the weight in the central pixel is 0.7 g minimum, with at least 1 adjacent pixel recording 0.3 g. This new version allows us to distinguish periods when the animal is static or dynamic by applying a mobility threshold of 700 ms—if each area in contact with the sensors is stationary for at least 700 ms, the animal is considered static, otherwise it is dynamic. Using the software, the operator then manually identified each paw (front left, front right, rear left, and rear right) and the areas in contact with the ground, which are identified as “Other zones” (tail, abdomen, head …) with the support of the video.

Each animal could move freely in the arena for 5 min in each pre-operative and post-operative session. The pre-operative session was recorded the day before the surgery, and then the time course of the syndrome was studied on days 1, 2, 3, 7, 10, 14, 17, 21, and 30 post-UVN (Figure 2).

### Data Analysis

The software performed a first automatic analysis of the acquisition file. This analysis involves the identification of the paw that activated each group of ground sensors. Following that, an experimenter checked the analyses and made corrections when necessary. Sequences where a paw cannot be clearly identified were removed from the analyses.

With this analysis, we could identify many parameters calculated automatically by the software (e.g., the weight distribution on each paw) as well as the support applied by each identified area of the animal's body in contact with the ground sensors (in grams) and the mean coordinates of these areas (in cm).

With the help of the software and home-made programs developed on Scilab (open-source software), different biomarkers were extracted, for static and dynamic phenotypes.

#### Static and Dynamic Parameters

The weight distributed on lateral axis during the acquisition allowed us to determine how the animal distributes its weight between his left and right paws in order to find its balance. This parameter had been investigated previously (6), but without distinguishing the static condition from the dynamic condition. This parameter was expressed as a percentage of the animal weight applied on the left limbs and right limbs at the day of acquisition.

By analogy with humans who use canes to gain postural stability, we tried to determine whether our rodent model had adopted a similar strategy by adding a support point on the ground. To that end, we quantified the average time the animals spent with their abdomen on the ground sensors, during static and dynamic periods. To do so, we calculated the time spent with another part of the body, with coordinates between the animal's four legs.

#### Dynamic Parameters

Circling is a phenotype specific to unilateral vestibular loss. This behavior happens when the rat starts to circle around its axis. We quantified this circling behavior by counting the number of fast laps performed during an acquisition. By tracking each front paw separately, we were able to quantify the deviation angle θ of each step made by the animal (Equations 1, 2). To do so, we used coordinates of the front paws during dynamic periods – FRx, FLx, FRy, and FLy are, respectively, the coordinates of the front right and front left paw on the X axis and on the Y axis. When the cumulative sum of θ was equal to 360° or −360°, it meant that the animal had made 1 complete lap with one paw, respectively, to the left or to the right. To distinguish the circling from the thigmotaxis (a specific behavior whereby the animal stays close to the walls of an arena during exploration), a 500 ms filter was applied over the duration of the supports of each leg. We then calculated the average of the number of laps obtained with the front left and right paws to quantify the number of laps made by circling during one acquisition.

(1) $$\begin{array}{l} \theta_{FR}=\text{ta}{\text{n}}^{-1}\left(FRy_{\text{n }+\;1},FRx_{\text{n }+\;1}\right)\;-\text{ ta}{\text{n}}^{-1}\left(FRy_{\text{n}},FRx_{\text{n}}\right) \end{array}$$

(2) $$\begin{array}{l} \theta_{FL}=\text{ta}{\text{n}}^{-1}\left(FLy_{\text{n }+\;1},FLx_{\text{n }+\;1}\right)\;-\text{ ta}{\text{n}}^{-1}\left(FLy_{\text{n}},FLx_{\text{n}}\right) \end{array}$$

#### Static Parameters

The support surface area is a sensitive parameter used to assess static postural instability in several models of unilateral vestibular loss [cats: (7), rats: (22)]. It was calculated by measuring the surface delimited by the four legs of rats while they were static during a session, using coordinates of each paws in the ground sensors. The maximum value for each acquisition was used to quantify instability moments.

##### Posturographic phenotypes

Previously, recorded data from the first version of this device (DWB® Bioseb SAS) were used to model the fine postural disruptions of UVN rats (6). To do that, the mean position of the barycenter was estimated with the help of the weight applied on each paw. Here, the use of paws coordinates allowed us to calculate a true barycenter of the bearing forces applied by the paws on the ground sensors (Equations 3, 4). The position of the barycenter was calculated at each period when the animal was stationary and on its four paws.

(3) $$\begin{array}{l} Barx=\frac{FLx*FLw+FRx*FRw+RLx*RLw+RRx*RRw}{FLw+FRw+RLw+RRw} \end{array}$$

(4) $$\begin{array}{l} Bary=\frac{FLy*FLw+FRy*FRw+RLy*RLw+RRy*RRw}{FLw+FRw+RLw+RRw} \end{array}$$

Based on the coordinates of the rat's barycenter over time, we were able to trace the statokinesigram of each acquisition. The statokinesigrams show the trajectories in 2D of the barycenter and the center of gravity of each paw every time the calculation is performed. The coordinates of the barycenter have also allowed us to use finer and more precise postural parameters already used in human clinical practice, such as the body sway area (body's balancing zone) [(15): Human, (13): Human, (23): mouse model]. These coordinates allowed us to quantify the postural stability and locomotion speed of our rat model as a function of the sway area (SFA) in order to estimate the energy spent by the rat to stabilize its posture (16). Indeed, on the same surface, the barycenter can cover a more or less long distance. This parameter is used in clinical posturology to estimate the energy a patient spent when he is on a force platform. In human clinical studies, the time the subject spends in a stationary position on a force platform is controlled. Here, we have chosen to use the speed of movement of the barycenter in order to avoid the variable durations of the static postures.

Body sway was evaluated by measuring the area of the confidence ellipse that includes 90% of the barycenter. With this classical method, we eliminate 10% of the extreme points to suppress postural sway values that were possibly due to quasi voluntary movements. This calculation was made when rats were static and on their four paws. An average weighted by the duration of each of these moments is then established for each acquisition.

### Statistical Analysis

For each of the parameters evaluated on the two groups of rats (UVN and SHAM groups) the recorded values are expressed as average + SEM. To test the effect of UVN, we performed analysis of variance (Two-way ANOVA with repeated measures). For *post-hoc* analyses, Dunnett's tests were performed to compare values at each post-operative time with pre-operative values, and Tukey-Kramer multiple comparison tests to compare the results obtained between UVN group and SHAM group and between static and dynamic conditions. The Tukey-Kramer multiple comparison test is adapted for comparison of samples of different size.

In order to avoid interindividual differences in postural parameters due to small differences in weight or foot placement between rats, all postural parameters were expressed as normalized data. Each result obtained by the animals was normalized with the result they individually obtained during the preoperative condition.

Pearson correlations were also calculated between the quantitative parameters and the parameters from the qualitative scoring scale in order to see or not similarities in their kinetics. These results give us the Pearson correlation coefficient r (no correlation if close to 0, low if between 0.3 and 0.5 and high if >0.5), and the significance of the correlation *p* (Table 1).

**Table 1:** Correlations between the results of qualitative behavioral assessment and the results of quantitative behavioral assessment of vestibular syndrome.

**Biomarkers used in the present study**	**Pearson r**	***P***
**Maximum support surface area**	**0.36**	0.0004*
**Time spent in the abdomen, static periods**	**0.3459**	0.0008*
**Time spent in the abdomen, dynamic periods**	**0.3542**	0.0006*
**Left circling**	**0.5592**	<0.0001*
Right circling	0.1504	0.157
Amount of energy spent to stabilize	0.1686	0.1121
Body sway area	−0.03676	0.7309
**Weight distribution on right limbs, static periods**	**0.3782**	0.0002*
**Weight distribution on left limbs, static periods**	**−0.3782**	0.0002*
Weight distribution on right limbs, dynamic periods	0.2149	0.042*
Weight distribution on left limbs, dynamic periods	−0.2149	0.042*

The parameters tested during this study are listed in the first column. In the second column the Pearson correlation coefficients r are listed, and in the third column their degree of significance. The parameters in bold are those correlated with the qualitative scale (low but significant correlations) (*p < 0.05).

## Results

This study analyses the longitudinal effects of a sudden and unilateral suppression of vestibular information on different posturo-locomotor biomarkers: [1] the weight distribution on the lateral axis and the time the animals spend leaning on their abdomen, in static and dynamic condition, [2] the number of turns performed in circling periods (behavior), [3] the maximum support surface area and posturographic parameters such as body sway and the amount of energy spent to stabilize (SFA), studied when the animal is static on its four paws. The results obtained in the UVN group are compared with preoperative values to observe the effects of the vestibular lesion, and with a SHAM group in order to avoid post-surgical effects or possible habituation effects to the task not visible under preoperative conditions.

### Static and Dynamic Parameters

#### Weight Distribution Along the Lateral Axis

**Figure 3:**
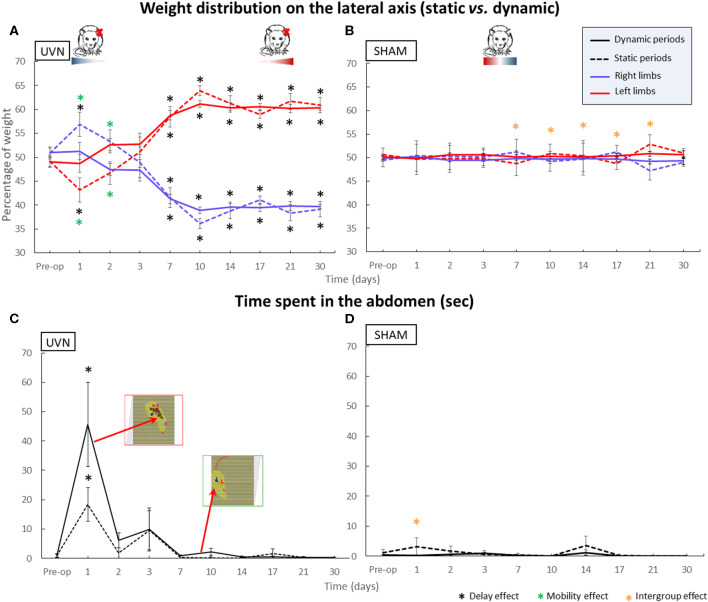
Parameters discriminating between static and dynamic behavior. Within A. The weight distribution on the lateral axis and in B. the time spent on the abdomen. **(A)** The weight distribution on the lateral axis shows asymmetric kinetics in the UVN group. Rats correctly distribute their weight between right and left limbs during the preoperative session and then switch more weight to their right limbs in static condition at day 1 & day 2 post-UVN (*p* < 0.05) while the dynamic weight distribution remains symmetrical. During the compensated period, UVN animals significantly distribute more weight on their left limbs whatever the static or dynamic condition. **(B)** SHAM animals always have a symmetrical weight distribution. The geometric shapes in color under the rat drawings indicate the amount of weight distribution on the left (red) and right (blue) paws in the UVN and SHAM group rats. **(C)** The UVN group spends significantly more time on their abdomen at day 1 postlesion compared to the preoperative session (0.0001 < *p* < 0.001) and compared to the SHAM group [static (*p* < 0.005) and dynamic (*p* < 0.0001)], with more time overall leaning on his abdomen in dynamic condition. **(D)** This behavior is never observed in the SHAM group. Standard errors of the mean are reported as vertical lines. Delay effect refers to significative differences with the preoperative condition; mobility effect means that a significative difference was found between static and dynamic condition; intergroup effect means that a significative difference was found between UVN and SHAM group (*p < 0.05).

The results for this parameter are shown in Figure 3. Before the unilateral vestibular lesion, the UVN group distributed its weight symmetrically between the right and left sides (static right % = 50.9 ± 1.3; static left % = 49.1 ± 1.3; dynamic right % = 51 ± 0.9; dynamic left % = 49 ± 0.9). At the first post-lesion day, no significant differences were observed on dynamic laterality parameters compared with preoperative values, or between the UVN group and the SHAM group. Nevertheless, in the UVN group, under static conditions, significant differences appeared between left and right limbs (*p* < 0.0001) and with the preoperative condition (*p* < 0.05). On the 2nd post-lesion day, significant differences still existed in the UVN group, in static condition, between left and right limbs (*p* < 0.05). No other differences were observed on days 2 and 3 post-lesion. From 1 week to 1 month after UVN, rats significantly shift their weights to the left limbs (D7: *p* < 0.001, D10–D30: *p* < 0.0001), with no difference between static and dynamic conditions. During this period, significant differences were also observed between the UVN group and the SHAM group (D7: *p* < 0.05, D10–D30: *p* < 0.0001).

During all post-lesion delays, under static and dynamic conditions, we can observe that the SHAM group had a symmetric weighting distribution between the right and left limbs (right % between 47.2 ± 2 and 51.2 ± 1.4 and left % between 48.8 ± 1.3 and 52.8 ± 2).

#### Time Spent Leaning on the Abdomen

Whatever the analysis time, the sham group never places its abdomen on the floor sensors as shown in Figure 3D. However, the abdomen of the UVN group animals is detected by the ground sensors from the first day after UVN (Figure 3C). At the first day post-UVN, significant differences are observed compared with the SHAM group (*p* < 0.005 and *p* < 0.0001 in static and dynamic condition; respectively) and with the preoperative condition for static (*p* < 0.001) and dynamic (*p* < 0.0001) conditions. During the 2nd and 3rd post-lesion days, some animals continue to lean on their abdomen for short periods of time but with no significant differences. The animals in the UVN group regain a posture without support on their abdomen from the 7th day postlesion.

### Dynamic Parameters

**Figure 4:**
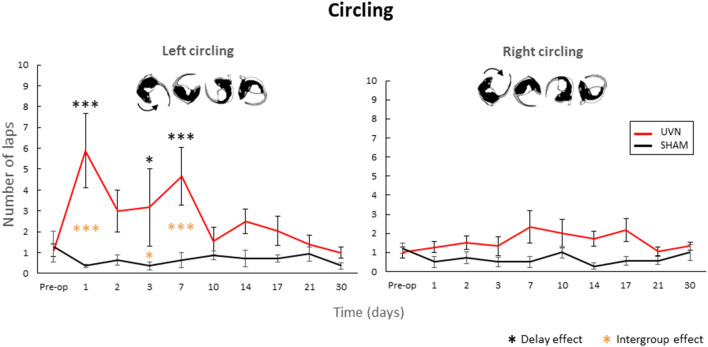
Quantification of the circling behavior. The UVN group performed significantly more left circling at D1, D3, and D7 compared to the preoperative period and the SHAM group (0.05 < *p* < 0.0001). Concerning the number of right-hand turns, no significant difference is observed between the SHAM and UVN groups. This circling behavior is never observed in the SHAM group. Standard errors of the mean are reported as vertical lines. Delay effect refers to significative differences with the preoperative condition; intergroup effect means that a significative difference was found between UVN and SHAM group (*p < 0.05; ***p < 0.001).

In order to have an objective quantification of the circling behavior observed in our vestibular syndrome model (5) and in several other disease models (24), we counted the number of complete turns performed with rapid leg support during all post-lesion days (Figure 4).

Concerning the number of right-hand turns, no significant difference is observed between the SHAM and UVN groups. The SHAM group performs on average <2 fast laps per session during all analyzed delays, and the UVN group performed a maximum of 2.3 ± 0.7 fast laps to the right at 7th day postlesion.

Significant differences are observed in the number of left laps circling at postlesion days 1, 3, and 7. These differences are observed between the UVN and the SHAM groups (D1: *p* < 0.0001, D3: *p* < 0.05, D7: *p* < 0.001) and with the preoperative values for the UVN group (D1: *p* < 0.0001, D3: *p* < 0.05, D7: *p* < 0.001).

### Static Parameters

#### Maximum Support Surface Area

The support surface area has been used in several animal models [(7) in cats, (22) in rats] to quantify postural instability following unilateral vestibular loss. Nevertheless, these data were acquired in the rat UVN model after reactivation of the vestibular syndrome by stimulation of the otolith system [(5) for more information]. Here, in order to quantify the postural instability, we have chosen to select the maximum surface area of the polygon during a session, which reflects instability periods of the animal.

**Figure 5:**
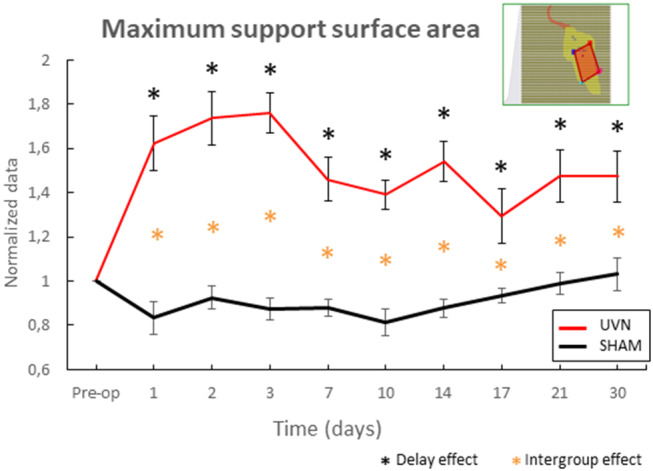
Automatic quantification of the maximum support surface area, a biomarker of postural instability. This parameter reveals instability following surgery in the NVU group at critical and compensated periods. Note that from the first post-lesion day to the last acquisition day, the maximum value of the support surface area of the sustentation polygon increases significantly in the UVN group compared to the preoperative time (0.05 < *p* < 0.0001), and compared to the SHAM group (0.05 < *p* < 0.0001). Standard errors of the mean are reported as vertical lines. Delay effect refers to significative differences with the preoperative condition; intergroup effect means that a significative difference was found between UVN and SHAM group (*p < 0.05).

The results obtained reveal a little variability in the SHAM group; the data normalized with the preoperative condition are between 0.8 and 1.2 (Figure 5). Nevertheless, from the first post-lesion day, the maximum value of the support surface area increases significantly in the UVN group compared both to the preoperative time (from day1 to day 7: *p* < 0.0001; day 10 and day 17: *p* < 0.05; day 14, day 21 and day 30 : *p* < 0.0001), and compared to the SHAM group (from day 1 to day 14 : *p* < 0.0001, from day 17 to day 30 : *p* < 0.05).

#### Posturographic Parameters

##### Statokinesiograms

**Figure 6:**
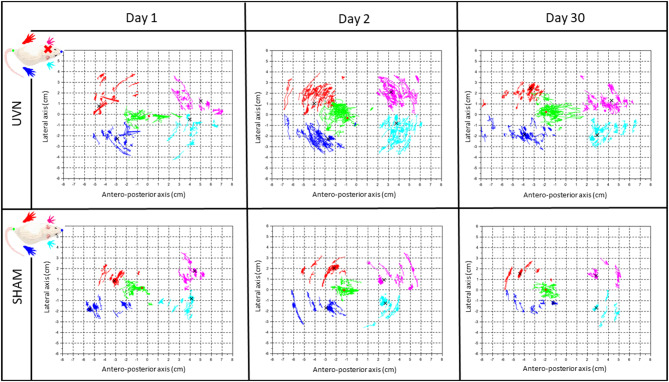
The rodent stabilogram, a representative illustration of the kinetics of barycenter and paws positions. Plot of the successive positions of the legs and the barycentre at each moment when the animal is stationary with its 4 paws placed on the sensors during a 5-min acquisition. The first line of the table gathers the stabilograms of a representative UVN rat at different post-UVN days (Day 1, Day 2, and Day 30), the second line gathers the stabilograms of a representative SHAM rat at these same post-UVN days times. For each stabilogram, the antero-posterior axis is on the abscissa and the lateral axis on the ordinate. The dark blue, red, light blue and pink dot clouds are the traces of the average positions, respectively, of the right rear paws, left rear paws, right front, and left front paws during a session at each moment when the animal is static on its four legs. The green point cloud is the trace of the successive positions of the barycentre calculated at each of these moments. The different black crosses represent the average position of the legs during an entire session. The red dot represents the average position of the barycentre during a session. The pattern of stabilograms appears stable in SHAM rats, compared to the UVN group where the pattern appears more instable at the three representative post-UVN days.

The calculation of the position of the barycenter at a frequency of 30 Hz, at each moment, when the animal is on its four paws and stationary, allowed us to trace the average positions of the paws and the position of the barycenter of each animal at each session. These statokinesiograms show us different postural patterns depending on the group of rats and the post-lesion time, including a greater dispersion of the barycenter point cloud at several post-lesion days (Figure 6). We then these plots to quantify the body sway and the energy expended to stabilize it (SFA).

##### Estimation of the energy spent to stabilize

**Figure 7:**
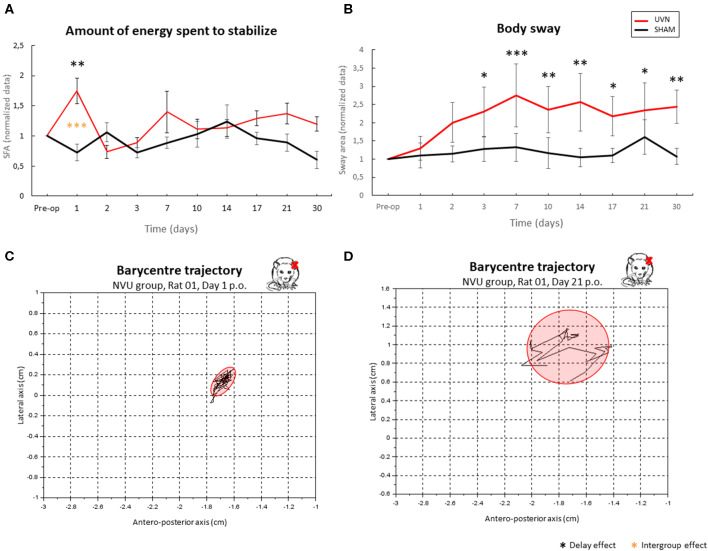
Use of posturographic parameters to quantify postural instability in a UVN rodent model. **(A)** quantification of the energy spent for posture stabilization. **(B)** body sway. **(C,D)** examples of barycentre plots observed in a representative NVU rat in acute **(C)** and compensated **(D)** period. The NVU group spends significantly more energy to stabilize on the first day post-UVN (*p* < 0.001); this energy is quantified by a ratio between the velocity of the barycentre and the surface of the confidence ellipse. It then stabilizes from D2 to D30 and becomes similar to that of the SHAM group, in which there is little variation at any time. The body-sway **(B)** is significantly higher in UVN rats from D3 to D30 and remains stable in SHAM rats. In **(C)**, we can see the representation of the displacement of the barycentre as well as its confidence ellipse at a time when the animal spends a lot of energy stabilizing its posture: the animal is stable but the displacement of its barycentre shows efforts spent to concentrate the position of its barycentre in the surface of the ellipse. In **(D)**, the same parameters are represented at a compensated delay: the animal is unstable (high surface area of confidence ellipse), and the dispersion of the path of its barycentre shows little effort applied to restrict the movement of the barycentre in a small area, that underlies a new postural strategy. Delay effect refers to significative differences with the preoperative condition; intergroup effect means that a significative difference was found between UVN and SHAM group (*p < 0.05; **p < 001; ***p < 0.001).

The results from the quantification of the energy expended to stabilize the posture (Figure 7A) show a significant difference on the first post-lesion day between the SHAM and UVN groups (*p* < 0.01) and with the preoperative condition for the UVN group (*p* < 0.001). No significant difference was observed at the preoperative time, and from the 2nd to the 30th post-lesion day between the two tested groups.

##### Quantification of postural instability

The results from the body sway (Figure 7B) show a significant gradual increase in this parameter beginning the 3rd postlesion day and persisting until the 30th postlesion day in the UVN group, indicating a progressive postural instability. The surface of the confidence ellipse at 90% of the barycenter doubles from postlesion D3 to postlesion D30 and remains significantly different with the preoperative time. No significant variation in this parameter is observed in the SHAM group, whose surface area of the normalized confidence ellipse remains close to 1.

### Statistical Correlations Between the Two Methods for Analyzing the Posturo-Locomotor Syndrome Following Unilateral Vestibular Suppression

**Figure 8:**
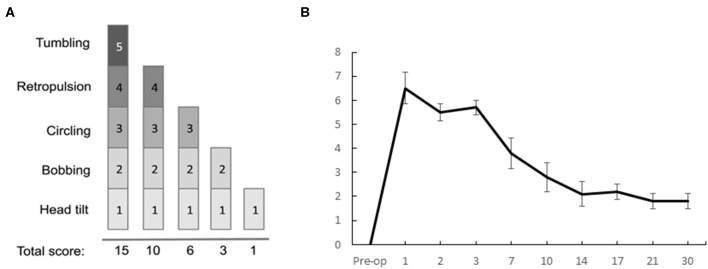
Qualitative evaluation of the posturo-locomotor components of the vestibular syndrome. **(A)** Illustration of the evaluation grid used to conduct the analysis. **(B)** Results of the qualitative evaluation of the UVN group at the different delays of the study: we can observe a critical period when the disorders are at their peak from D1 to D3, then a period when the disorders gradually compensate each other, with a progressive decrease in the score from D7 to D30.

The results from the qualitative vestibular syndrome assessment scale used in the same rodent model [(5), Figure 8A] describe the kinetics of vestibular syndrome. The disorders are expressed at their peak during the first 3 days after UVN, then gradually decrease at the 7th post lesion day to stabilize at a score of 2 on average at the 30th postlesion day (Figure 8B).

Correlations were made between the quantitative and qualitative parameters in the UVN (Table 1). Low (0.35 < r < 0.38) but highly significant (*p* < 0.001) correlations exist between the results of the qualitative scale and the quantitative parameters: maximum support surface area, time spent in the abdomen (static and dynamic periods), weight distribution on left limbs (static periods). The left circling quantitative parameter is strongly and significantly correlated with the qualitative scoring (*r* = 0.56; *p* < 0.0001). The weight distribution quantitative parameter on the left legs is inversely correlated to the qualitative scale (*r* = −0.38; *p* < 0.0002).

## Discussion

This study provides new information on the postural balance patterns observed in a rodent model of acute vestibular peripheral pathology (UVN rat model). We quantify for the first-time postural parameters, such as those analyzed in human clinical studies in the field of posturology. Plots of the position of the animals' barycenter under static conditions (stabilograms), the surface of the 90% ellipse of confidence of these stabilograms, the speed of displacement of the barycenter, and the speed of the barycenter as a function of the sway area (SFA) provide estimates of the energy used for postural stabilization. In addition, two new vestibular behavioral phenotypes are also quantified for the first time in this study: the time spent by the animal leaning on its abdomen and the number of laps performed during the circling behavior. This was done through a newly automated and parametric analysis method that is independent but correlated with the qualitative scales that are traditionally used (4, 5, 25).

These new postural biomarkers have allowed us to differentiate static behavior from dynamic behavior, to automatically quantify circling in a unilateral vestibular lesion model (behavior usually noted in qualitative score scales), and to focus precisely on the postural disorders inherent to our rodent model of unilateral vestibular pathology.

### Static and Dynamic Behavioral Parameters

Based on the impact of vestibular loss on the weight distribution on the lateral axis, we have suggested a strategy for postural rebalancing during the acute phase of vestibular syndrome. Indeed, at D1 and D2 postlesion, animals exhibit good dynamic balance because they distribute their weight symmetrically, whereas this distribution is asymmetric with more weight applied on the ipsilesional side under static conditions. The control of locomotion is managed by a very large network (26). These neural networks are located in the spinal cord and form the central pattern generators (CPGs) of locomotion. These CPGs, through the action of a pacemaker neural network (27), are able to initiate rhythmic motor control in the absence of sensory feedback. Rats have 2 CPGs: one at the cervical level for the control of the rhythmicity of the front limbs and one at the lumbar level for the control of the rhythmicity of the movements of the trunk and hindlimbs (28). Thus, once motor control is initiated and in the absence of external disturbances, the CPGs generate an automatic rhythm for walking. Under static conditions, the information obtained from the hair cells of the peripheral vestibular receptors located mainly in the saccule and utricle becomes essential to maintain anti-gravity muscle tone during rest (29). Thus, during the acute phase of vestibular syndrome (the critical period of 1–3 days postlesion), when the animal is stationary, UVN causes asymmetry in the anti-gravity muscle tone, with a decrease on the ipsilateral side due to the absence of gravity information from the deafferented side. This asymmetry in muscle tone is probably responsible for the larger weight distribution on the right-side paws and the use of the abdomen for support as a postural stabilization strategy. Weight rebalancing under dynamic conditions can also occur due to the contribution of other sensory modalities, thus supporting the sensory substitution phenomenon observed in patients after vestibular neurectomy (13). Indeed, during locomotion, plantar information and visual flow can augment the sensory inputs and thus contribute to the rebalancing of electrophysiological activity between the two vestibular nuclei (VN), which is known to be the key parameter of vestibular compensation (30). During locomotion, the activity of spinal cord CPGs take control of the locomotor system and also contribute to the restoration of electrophysiological homeostasis of the VN since these spinal neural networks project directly onto the VN (31) (Figure 1A). This postural readjustment is also observed under dynamic conditions in patients. An increase in walking speed leads to a decrease in the gait deviations observed in vestibular patients (32).

In addition, the data from the UVN group's abdominal exposure time indicate that during this critical period (from 1 to 3 days postlesion), animals place their abdomen on the ground sensors. This behavior was not observed during the other postlesion days or in the SHAM group. We can also observe that they use their abdomen as a support, especially in dynamic conditions. We hypothesize that UVN rats use their abdomen to maintain dynamic balance, and this part of the body acts as a new support point used to promote stability, especially under dynamic conditions. The same type of behavior is found in humans, as people with poor balance tend to lean on a cane.

From the 7th day postlesion, the animals distribute much more weight on the ipsilesional side, which is consistent with the results previously published (6) and reflects muscle tone recovery on the ipsilesional side. Nevertheless, the animals in the UVN group maintained this weight distribution asymmetry on the lateral axis until the 30th day after UVN. It can be assumed that this weight asymmetry in favor of the injured side may explain the body's inclination and the deviation of the locomotor trajectory on the side of vestibular loss, as observed in patients after UVN (13, 32, 33).

It can also be noted that this weight asymmetry appears as early as 7 days postlesion, which coincides with the time at which the animals stopped the abdomen, support on the ground sensors. Although the animals no longer used their abdomen because they recovered muscle tone in their limbs, their weight redistribution pattern on the lateral axis was different from that observed in the preoperative period. The animals were able to compensate but not fully recover to preoperative levels after experiencing vestibular loss.

### Dynamic Parameter

Circling refers to rapid rotational behavior that is observed in different disease models (4, 5, 7, 24). This phenotype is common to rodent models of various pathologies with cerebral asymmetry (Parkinson's disease, schizophrenia, depression or anxiety). A similar behavior has been observed in humans: in the absence of visual information, they exhibit a circular deviation to the right or left in their locomotor pattern (34). The authors of this article suggest that this phenomenon may be related to an asymmetry in non-pathological vestibular information.

In the literature, the most common hypothesis explaining circling is a dopaminergic imbalance in the striatal pathways (19). However, the VN project massively toward the thalamic parafascicular nucleus, which in turn projects directly toward the striatum (Figure 1A). It is recognized that this vestibulo-thalamo-striatal pathway is essential for the control of posture and leg movements (35). Thus, circling may be a result of a striatal electrophysiological imbalances resulting from electrophysiological imbalances that are observed in the VN after unilateral vestibular loss (Figure 1B). Similarly, its disappearance may be linked to a rebalancing of the electrical activity in the striatum. Indeed, it is well-demonstrated in the literature that vestibular syndrome is the result of electrophysiological asymmetry between homologous VN, characterized as low spontaneous electrical activity on the deafferented side and high activity on the intact side. Studies in the literature also indicate that restoring the electrophysiological balance between the two opposing VN is the key parameter for postural, locomotor and gaze stabilization function recovery (30, 36–41). In addition, the reticulospinal pathway is modulated by vestibular, visual and proprioceptive information. According to the model proposed by Deliagina et al. (42), UVN causes an imbalance in the electrophysiological activity recorded in the reticular formation, resulting in locomotor imbalances, which has been shown to result in rolling behaviors in lamprey. Circling behavior may thus represent a behavioral phenotype typical of an alteration in the excitation vs. inhibition balance of a heterogeneous neural network including, among others, VN, the striatum, the reticular formation and locomotor CPGs.

### Static Parameters: Posturology

The support surface area is usually measured in animal models of vestibular pathology to assess static postural deficits following unilateral vestibular lesions (7, 22). In rats, this parameter is usually used in situations in which the syndrome is reactivated. In the present study, the support surface was calculated in a spontaneous situation of instability (the maximum value per session was selected). Our results show that the area of the support surface became significantly larger and reached the maximum size during the acute period, which is 1–3 days postlesion. This area gradually decreased but remained significantly wider postoperatively compared with preoperatively. These results indicate persistent postlesion postural instability but provide little information on the fine kinetics of vestibular syndrome. Posturology assessments provide more information about the static deficits observed in our rodent model. This used in human clinical practice for the diagnosis and evaluation of postural disorders and for the assessment of instability. Statokinesiograms are shown here for the first time in rats under ecological conditions, without any restraint, unlike in other studies (23, 43). In this study, postural parameters are automatically studied when an animal voluntarily immobilizes itself, which reduces the need to retrain animals subjected to a stressful model of pathology (25). The analysis of the barycenter was carried out when the animal was at rest on its 4 paws, and 10% of the extreme values were excluded so that the barycenter positions where the animal started a voluntary movement were not included in the dataset.

Body sway was also analyzed for the first time in an automated way in rats, and the results indicate that instability increases gradually after vestibular loss, reaching a peak at D7, and remains high until 1 month after UVN. These data are comparable to those obtained in patients after the same type of vestibular lesion (UVN), which show an increase in body sway that is maintained over time (13). In another study, Deveze et al. (14) observed posturographic deficits in vestibular patients, which were also maintained over the long term without rehabilitation. In addition, a similar analysis showed that body sway is a good posturo-locomotor phenotypic biomarker when used for the differentiation of different neurodegenerative diseases (23).

The energy expended for postural stabilization increases sharply on the first day after UVN and then normalizes. It seems logical that the level of energy expenditure is highest during the peak of the severity of vestibular syndrome. According to the qualitative analysis, the postural disorder is the most severe on the first day postlesion, when behaviors such as circling and tumbling are observed (5, 6). Restoring postural balance to compensate for these deficits certainly requires a considerable amount of energy and effort. The energy parameter has never been studied in animals subjected to unilateral vestibular loss. A study in vestibular patients using the wavelet method showed the same results; vestibular loss patients spent more energy maintaining balance than control subjects (12). This parameter is therefore a good tool to assess the difficulty of an animal suffering from postural disorders of different origins in maintaining balance.

The posturo-locomotor deficits observed in the acute phase are probably due to the excitability imbalance between the two homologous VN (Figure 1B). It is the electrophysiological asymmetry between VN that induces an asymmetry in muscle tone that is responsible for postural imbalances. Conversely, it can be assumed that the restoration of certain postural biomarkers (time spent on the abdomen, circular behavior, SFA) in the period in which compensatory behaviors are developed results from the restoration of electrophysiological homeostasis between the VN (21). Nevertheless, changes in some biomarkers (ipsilateral weight distribution, body sway) lead to the development of a new postural strategy expressed during the period in which compensatory behaviors are developed. These biomarkers cannot be estimated by an experimenter and have therefore never been included in the qualitative assessment scales for vestibular syndrome. Persistent long-term postural locomotor disorders have also been clinically identified in vestibular patients.

In this study, we provide the results of a new way of quantifying the posture-locomotor deficits associated with vestibular lesions and the compensatory strategies adopted. We highlighted new parameters that can be considered finer biomarkers of postural and locomotor alterations following vestibular loss. These data corroborate the phenomenon of vestibular compensation, which is well-described in the literature, and also highlight the presence of persistent balance disorders in vestibular lesioned rats. The focus of pre-clinical therapies should therefore be to resolve these persistent disorders in rodent models, thus leading to improvements in postural balance during the period in which compensatory behaviors are developed. This parametric approach is more sensitive than traditional methods in the evaluation of unilateral vestibular syndrome in rodents, so it is anticipated that it will become an essential evaluation tool used to test the efficacy of anti-vertigo compounds or rehabilitation protocols on the kinetics and the quality of restoration of posture-locomotor balance after vestibular loss.

## Data Availability Statement

The datasets generated for this study are available on request to the corresponding author.

## Ethics Statement

The animal study was reviewed and approved by French Agriculture Ministry Authorization: B13-055-25. Neuroscience Ethic Committee N°71 from the French National Committee of animal experimentation.

## Author Contributions

EM and BT: conceptualization and writing—original draft. EM, NE, and GR: data curation. EM, VA, DP, and BT: formal analysis. BT and CC: funding acquisition. EM, BT, NE, GR, DP, and VA: investigation. EM, BT, DP, and VA: methodology. BT: project administration. BT and VA: supervision and validation. EM, BT, and CC: writing—review & editing.

## Conflict of Interest

The authors declare that the research was conducted in the absence of any commercial or financial relationships that could be construed as a potential conflict of interest.
